# Editorial: Structure-Based Drug Design for Diagnosis and Treatment of Neurological Diseases

**DOI:** 10.3389/fphar.2017.00013

**Published:** 2017-01-24

**Authors:** Rona R. Ramsay, Giuseppe Di Giovanni

**Affiliations:** ^1^Biomedical Sciences Research Centre, School of Biology, University of St AndrewsSt Andrews, UK; ^2^Department of Physiology and Biochemistry, University of MaltaMsida, Malta

**Keywords:** neurodegenerative diseases, medicinal chemistry, pharmacology, data-mining, QSAR, monoaminegic multi-target drugs, epliepsy, Alzheimer's disease

Written by researchers brought together by COST Action CM1103 to address multi-target drug design for the complex challenge of neuropathology, the interdisciplinary reviews span the range from computational enzymology through medicinal chemistry and pharmacology to human studies.

To introduce the process of designing drugs that hit the multiple targets identified as important complex neuropathologies, Hughes et al. present a brief overview of the current approaches of approaches of data-mining, *in silico* screening, and rational drug design that rely heavily on clinical and biological validation of suitable targets and the molecular understanding of these targets by their crystallographic structures. Computational advances have been particularly important in the last 5 years, tackling the workload required for multiple targets. Nikolic et al. used data-mining to search for off-target activities, followed by docking compounds to targets to assess their potential as ligands, to provide new leads for experimental confirmation and optimization. Through cheminformatic, 3D-QSAR and virtual screening methodologies, ligands were identified for the traditional neurotransmitter breakdown enzymes, cholinesterases and monoamine oxidases, that also bind to combinations of dopamine, serotonin and histamine receptors, a vital contribution to efficiency in the rational design of multipotent ligands with selected polypharmacology. The next article moves into molecular dynamics on receptors where ligand binding can increase or decrease the response of the receptor, and on to ion channels and their conformational changes responsible for gating. Vianello et al. also review the investigations on the critical chemical step in the inactivation of monoamine oxidase B by selegiline, using a combination of the high-resolution quantum mechanical calculations with molecular mechanics (multiscale QM/MM simulation). Reviewing a hotly-debated topic, the article gives a clear exposition of the evidence for a hydride mechanism for monoamine oxidases.

Although computational methods have accelerated compound discovery, lead compounds must still be synthesized. Marco-Contelles et al. describe the optimization of a compound designed to inhibit multiple targets in Alzheimer's disease, showing the derivation of ASS234 from single-target ligands and its assessment, including inhibition of cholinesterases and monoamine oxidases, antioxidant properties, low toxicity and suitable permeability. *In vivo*, ASS234 proved to be neuroprotective and to prevent β-amyloid aggregation in a mouse model. The medicinal chemistry theme continues with two articles on drug design for receptors. Khanfar et al. focus on combinations with antagonists of the histamine H3 receptor. H3R plays an important role as a hetero-receptor in the modulation of the release of other neurotransmitters including acetylcholine, noradrenaline, dopamine, GABA, glutamate, and serotonin. Thus, antagonists may have a therapeutic role in preventing negative feedback on neurotransmitters known to be involved in depression, schizophrenia, and Alzheimer's Disease. The authors discuss the challenges and target choice (other receptors, transporters, or enzymes such as histamine N-methyl transferase) in designing combinations for these receptors. Maramai et al. explore the therapeutic importance of D3 antagonists or partial agonists and examine possible combinations that would be useful in the dysregulation of the dopamine neurotransmitter system linked to schizophrenia. A complexity in multi-target design for these G-protein coupled receptors is that many of them form heterodimers (such as H3 with D2, mentioned in Khanfar et al.). In particular, D3 receptors can form homodimers or heteromers with D1 or D2 receptors, modulating the response to ligands. The authors then review recent compound discovery on a variety of innovative scaffolds for attaining high D3 receptor affinity and selectivity over the D2 receptors.

The next two articles address molecular pharmacology. Unzeta et al. consider how one specific multi-target designed ligand (MTDL) addresses a selection of the multiple possible targets in Alzheimer's disease. ASS234 was designed from donepezil, a cholinesterase inhibitor used in the clinic, and an inhibitor of monoamine oxidase B, of the same propargyl type as deprenyl. Derivatives of ASS234 were designed to improve affinity to the enzyme targets and to incorporate metal ion binding and antioxidant properties, two important targets in preventing cell death in the brain. The pharmacology of the targets is also reviewed to explain why action at these targets is needed. In particular, the role of metal ions and of inflammation in brain pathology is reviewed as these are more complex target areas than enzymes and receptors. Ramsay et al. consider the biochemical pharmacology used to evaluate the new multi-target compounds against the accepted enzyme targets, including cholinesterases and monoamine oxidases, and then review new targets in mitochondrial function. Advances in cell biology have demonstrated that mitochondrial fission and fusion is a protective mechanism in cellular bioenergetics. Regulation of these processes is therefore emerging as a target for new therapeutic strategies to prevent neurodegeneration. Finally, drug metabolism by the cytochrome P450 system, a vital part of any compound evaluation, is also reviewed.

Moving on to *in vivo* work, Di Giovanni et al. discuss neuropharmacological studies on treatments for and novel compounds designed to tackle schizophrenia, depression, and obesity, conditions where monoamines have been implicated. The monoamine pathways include dopaminergic neurons in the ventral tegmental area and the substantia nigra pars compacta, noradrenergic neurons arising from locus coeruleus, serotonergic neurons originating from median and dorsal raphe nuclei, and histaminergic neurons from the hypothalamus. The authors use an excellent figure to summarize the biosynthesis, metabolism, receptors and transporters of these systems, before discussing known drugs and why consideration of the monoamine systems is important in neurological conditions such as epilepsy, Alzheimer's Disease and stroke. Epilepsy is considered in more depth by Svob Strac et al., who propose that monoaminergic multitarget drugs could provide a therapeutic opportunity. Drawing from experimental, clinical, and genetic studies in animals and humans, they consider each of the monoamine systems, summarizing the evidence to identify key proteins as targets in epilepsy providing a basis for future monoaminergic strategies to treat epilepsy by recognizing it as an imbalance of brain chemicals and physiology due a multitude different mechanisms, requiring multi-target drugs.

In conclusion, to identify successful multi-target compounds, extensive pharmacological characterization is essential, as is the ability to learn about the drug-target interactions from the large amount of data being generated. However, which combinations of targets will be effective is still a big question.

## Author contributions

RR drafted the editorial; both authors revised and approved it.

### Conflict of interest statement

The authors declare that the research was conducted in the absence of any commercial or financial relationships that could be construed as a potential conflict of interest.

